# 40. Rivaroxaban for treatment of vascular BehÇet's disease

**DOI:** 10.1093/rap/rky034.003

**Published:** 2018-09-20

**Authors:** Reem Musa, Loay Al Dhahir

**Affiliations:** Internal Medicine, Dr Sulaiman Al Habib Hospital, Riyadh, SAUDI ARABIA

**Introduction:** Treatment of venous thrombosis in Behçet's disease (BD) has always been a grey area in medical practice. The authors describe the first treated case of recurrent multiple venous thrombosis (VT) in BD with rivaroxaban in Saudi Arabia. The patient has been successfully treated during the last four years with rivaroxaban without any complications or new VT features. We report this case hoping to start research for new line of management for this condition, which will help other patients for better compliance and efficiency as well as improving the quality of life using novel oral anticoagulants (NOACs). BD (traditionally known as Silk Road disease) is a rare systemic chronic condition with relapsing remitting vasculitis of unknown aetiology. It affects both arteries and veins. Clinical traits include oral and genital ulcers, ocular involvement including anterior and posterior uveitis, and retinal vacuities, which can lead to visual loss if not assessed and treated carefully. The disease mainly manifests as mucocutaneuos disorder but can involve any other systems, multifactorial aetiology has been hypothesised including HLA-B51 and HLA-ERAP-1 genes, but the exact aetiology has not yet been identified. BD has the highest frequency in patients between 20-40 years old, but can also be seen in children and the elderly. Sex distribution is variable but incidence is higher in males in high prevalence areas (Turkish and Middle East populations). The disease is mainly sporadic but can be seen in families. Vascular manifestations are present in up to 40% of the patients with BD with a high rate of recurrence. Refractory thrombosis has been observed to be one of the presenting sign at the time of diagnosis. Although there is no link yet between thrombophilic disorders and BD, patients with this disease have a considerable rate of VT and relapse.

**Case description:** A 33 year old male was diagnosed with BD, when presented to our outpatient clinic with the chief complaint of recurrent oral and genital ulcers. The patient was commenced on standard immunosuppressives (azathioprine and colchicine), however during the follow-up period he developed a deep vein thrombosis (DVT). The decision was made to start oral anticoagulant with vitamin K antagonist agents (VKA); unfortunately, the patient had poor compliance, with erratic INR complicated by bleeding once in addition to multiple presentations to ER and outpatient clinic with recurrent DVT. During one of these episodes, the patient presented with altered visual acuity, and was diagnosed to have left eye retinal vein thrombosis which further deteriorated to complete visual loss. The patient has no family history of BD, other thromboembolic or vasculitic disorders. Unexpectedly, he presented again with DVT despite being on VKA and low molecular weight heparin (LMWH) most likely due to poor compliance, then rivaroxiban was discussed as an alternative treatment. The patient informed about the uncertain use of this medication, side effecst and the fact that there is no evidence of its effectiveness in treating or preventing VT in his disease. Patient commenced on rivaroxaban with satisfying outcome for four years of regular follow-up with no complications or features of vascular thrombosis. The compliance improved as patient felt more comfortable without having repeated INR monitoring. The patient has had an uneventful clinical course after starting rivaroxaban.

**Discussion:** This is the first known case of BD in Saudi Arabia where the frequency of venous thromboembolic events were successfully managed with rivaroxaban over a course of over four years of outpatient follow-up. BD has a high prevalence in the Mediterranean, and considerable prevalence in others. Vascular BD is unique in affecting both arterial and venous systems and is a major cause of morbidity and mortality, despite there being no clear guidelines for the treatment and secondary prevention of VBD. Immunosuppressive agents have demonstrated significant reduction of venous thromboembolic events in BD; however, in clinical practice we often encounter patients with BD who have recurrent VT while on immunosuppressant medications, and oral VKA (warfarin) with the commonest reason cause being sub-therapeutic drug levels due to poor compliance or intolerance. Rivaroxaban has a fixed dose and doesn’t require blood monitoring. Moreover, it has limited drugs interactions. It has been proven to be effective, safe and has significant impact in decreasing the recurrence of VT with lower risk of gastrointestinal bleeding and subsequent complications comparing to VKA in situations other than BD. Patients with DVT treated with rivaroxiban have a lower rate of hospitalisation and outpatient visits without increasing the risk of readmission. Rivaroxaban has shown a great result in treatment and secondary prevention in this patient, so this might open a new line of treatment and secondary prevention of VBD if proven in further clinical trials.

**Key Learning Points:** BD is rare but has serious complications, especially vascular issues, if not detected and treated carefully. There are no new guidelines in the treatment of BD. Although immunosuppressive agents proved reduction in vascular complications in BD, we still face patients with venous thrombosis despite being on immunosppressant medications. Rivaroxaban might be a new line in treatment and secondary prevention of vascular BD if proven in further clinical trials.

**Disclosure: R. Musa:** None. **L. Al Dhahir:** None.

